# Choosing a COVID-19 vaccination site might be driven by anxiety and body vigilance

**DOI:** 10.1515/med-2024-0958

**Published:** 2024-05-20

**Authors:** Maja Simonović, Nikola M. Stojanović, Sonja Novak, Mirjana Radisavljević, Olivera Žikić, Tatjana Milenković

**Affiliations:** Department of Psychiatry, Faculty of Medicine, University of Niš, 18000 Niš, Serbia; Center for Mental Health Protection, Department for Diagnose and Treatment, University Clinical Center, 18000 Niš, Serbia; Department of Physiology, Faculty of Medicine, University of Niš, 18000 Niš, Serbia; Department of Epidemiology, Public Health Institute, 18000 Niš, Serbia; Clinics for Gastroenterology, University Clinical Centre Niš, 18000 Niš, Serbia

**Keywords:** COVID-19 vaccine, anxiety, body vigilance

## Abstract

**Background:**

The occurrence of COVID-19 led to the rapid development of several vaccines which were distributed around the world. Even though there had been a vast amount of information about both virus and vaccination, this process was potentially related to increased anxiety and thus affected the vaccination process.

**Objective:**

The present study examined anxiety levels and body vigilance in subjects reporting for COVID-19 vaccination at different vaccination sites.

**Methods:**

Instruments used included general socio-demographic questionnaires and specifically constructed ones such as generalized anxiety disorder (GAD), body vigilance scale (BVS), and coronavirus anxiety scale (CAS).

**Results:**

A total of 227 subjects enrolled in the study reported mild GAD and CAS scores and relatively low scores on BVS. When the subjects were divided according to a vaccination site (under supervision and non-supervised), it turned out that subjects vaccinated under supervision were more anxious (higher GAD and CAS) and had their body vigilance increased.

**Conclusion:**

In conclusion, there is a need for highlighting the importance of efficient planning and organization of vaccination process, since to a certain extent it is driven by both anxiety and body vigilance.

## 1 Introduction

The rapid development of a vaccine accompanied the emergence of the COVID-19 pandemic [1] as an opportunity to prevent and protect against severe forms of infection and potential complications [2]. Also, the vaccination process could have influenced the financial burden that every country experienced during the pandemic, including Serbia [3]. Activities related to the need for widespread use of vaccination have become a necessity and an obligation for all medical staff. The question arose as to how to motivate people to make a decision and get vaccinated in accordance with the recommendations of professionals. The need for careful observation and analysis of the effects of the procedure accompanies the vaccination of many people with a new vaccine. Furthermore, vaccination must achieve sufficient equality between all countries for the world to return to a pre-pandemic status [2]. A recent study conducted on the Serbian population suggested that there are five main reasons for COVID-19 vaccine hesitancy, which include concern over vaccine side effects, concern over vaccine effectiveness, concern over insufficiently tested vaccines, mistrust of authorities, and conspiracy theories [4]. The concern related to the vaccine side effects might be overcome by the vaccination of people under strict medical staff supervision in the emergency departments.

The majority of publications to date are focusing on the impact of the COVID-19 pandemic on mental health [5,6]. However, not much attention has been paid to identifying predictors of COVID-19-related anxiety [7]. COVID-19-related anxiety can vary among individuals and may be influenced by a range of factors. Although these factors are pretty individual, some could be described as general and include pre-existing mental health conditions, health concerns, media exposure, perceived risk, social isolation, etc. [8]. It is essential to recognize that these factors can interact and vary among different populations and potentially distinguish them if they arrive from somatic diseases, general anxiety, and sensitivity to interoceptive stimuli or fear due to the pandemic. This kind of presumption is evident from the fact that individuals with pre-existing mental health conditions, such as anxiety disorders or depression, are more susceptible to experiencing heightened anxiety during the pandemic [9].

While common intuition implies that perception arises after sensation, indicating that bodily feelings have their origin in the body, contemporary evidence challenges this notion. Recent findings propose a different perspective: interoceptive experiences might predominantly result from limbic system predictions regarding the anticipated condition of the body. These predictions are then influenced by ascending visceral sensations. Thus, diverting from the traditional view that bodily feelings are solely a consequence of sensory input from the body [10,11]. Thus, if we accept this perspective, one might argue that general anxiety, with its origin in the limbic system [12], might influence bodily feelings.

The present study aimed to determine the anxiety levels and body surveillance in individuals who reported for COVID-19 vaccination at different vaccination sites and to compare the findings. Thus, using three instruments, we evaluated the presence of somatic disorders, anxiety and sensitivity to bodily sensations, and anxiety due to the COVID-19 pandemic.

## 2 Materials and methods

### 2.1 Subjects and data collection

The present research was designed as a prospective study conducted at the Vaccination site located at The University Clinical Center Nis (under medical staff supervision) and at the Local Health Center Dom zdravlja Nis (control) between March and August 2021. The prospective study collected the data after the approval of the Ethics committee from the beginning of March 2021 until August 2021. All subjects reporting to the vaccination were initially approached with a suggestion for participation, and only those who accepted were further enrolled. All enrolled subjects were over 18 years of age. Subjects were first given to complete socio-demographic and vaccination questionnaires, and only after that did they receive three questionnaires about their anxiety and body vigilance.

### 2.2 Instruments used

#### 2.2.1 Generalized anxiety disorder-7 (GAD-7)

The GAD-7 questionnaire comprises seven items designed to assess symptoms of worry and anxiety. Each item is rated on a four-point Likert scale (0–3), with response categories ranging from “not at all” to “nearly every day.” Total scores on the GAD-7 can range from 0 to 21, with higher scores indicating greater severity of anxiety symptoms. Cut-off points are established at 5, 10, and 15 scores, denoting mild, moderate, and severe anxiety levels, respectively. However, scores exceeding 10 are considered within the clinical range, as suggested by Spitzer and co-workers (2006). The GAD-7 has demonstrated reliability and construct validity in previous research [13]. Cronbach’s alpha for the GAD-7 was 0.91.

#### 2.2.2 Coronavirus anxiety scale (C-19ASS - CAS)

The CAS is a nine-item instrument designed to measure anxiety specifically linked to COVID-19, and they were found to correlate with the presence of perseverate thinking (six items) and avoidance (three items). The items evaluate the frequency of anxious responses related to the coronavirus, such as “I have avoided using public transport because of the fear of contracting coronavirus (COVID-19)” over a period of last two weeks. Respondents rate (Likert scale) items on a scale from 0 (not at all) to 4 (nearly every day), with total scores ranging from 0 to 36. The C-19ASS has demonstrated good internal consistency and test–retest reliability [14]. In the current study, Cronbach’s alpha for the CAS was 0.92.

#### 2.2.3 Body vigilance scale (BVS)

The BVS is a concise four-item measure designed to evaluate an individual’s inclination to attend to arousal-related body sensations closely. The initial three items gauge attentional focus, sensitivity to changes, and time dedicated to monitoring body sensations. Respondents rate these aspects on a scale ranging from 0 (not at all) to 10 (extremely). In the fourth item, respondents individually assess the level of attention directed towards 15 specific body sensations (e.g., heart palpitations) on a scale of 0 (none) to 10 (extreme). These individual ratings are then averaged to generate a single score. Possible scores on the BVS range from 0 to 40, with lower scores indicative of lower levels of body vigilance. The BVS has shown good internal consistency and test–retest reliability [15], and in the present study, Cronbach’s alpha for the BVS was 0.94.

### 2.3 Statistical analysis

Numerical data are presented as mean ± standard deviation. Comparison of values between two groups was performed by either Student’s *t*-test or Mann–Whitney test in accordance with data distribution. Categorical variables were reported as the Chi-square test made counts, percentages, and between-group comparisons. Adjusted residuals were used to evaluate associations between categorical variables and outcomes. The null hypothesis was tested with a significance threshold of *p* < 0.05. All statistical analyses were performed using R software (version 3.0.3) (R Foundation for Statistical Computing, Vienna, Austria).


**Informed consent:** All participants provided written informed consent before participating in this study. The confidentiality of participants’ personal information was strictly maintained, and data were anonymized and stored securely. Participation in this study was voluntary, and participants could withdraw at any time without consequences. This study posed no physical or psychological harm to the participants, and appropriate measures were taken to ensure their well-being throughout this procedure.
**Ethical approval:** This study was conducted following the ethical principles outlined in the Declaration of Helsinki [16]. Ethics Committee approval was obtained from the institutional review board of the University Clinical Centre Niš and Local Health Center Nis (17621/16 and 2307, respectively, both from 2021).

## 3 Results

A total of 227 respondents were included in the research, with a mean age of the studied population 43.6 ± 12.4. In the surveyed population, 34.8% of respondents were male, 82.4% were respondents from the city, and 48.6% of respondents were with high school education. Previous occurrence of allergies was present in 49.3% of respondents, while chronic diseases were present in 33.9% of respondents (Table 1).

**Table 1:** Demographic and clinical characteristics of the study population

Parameter	Mean ± SD1 or *n* (%)
Age	43.6 ± 12.4
Gender – male	79 (34.8)
Location	
City	40 (17.6%)
Rural area	187 (82.4%)
Education level*	
Primary	6 (2.6%)
Secondary	106 (48.6%)
Post-secondary	33 (15.1%)
Tertiary	73 (33.5)
Previous allergies	112 (49.3%)
Chronic diseases	77 (33.9%)
Cardiovascular	42 (18.5%)
Diabetes mellitus	14 (6.2%)
Endocrinological	12 (5.3%)
Kidney disorders	4 (1.8)
Oncological patients	3 (1.3%)

*Nine subjects did not fill the education level.

In the subsequent analysis, subjects were divided based on the vaccination site where they recruited and filled the questionnaires. Out of the total number of subjects, two subgroups were formed. One group served as a control (*n* = 95) consisting of subjects who were vaccinated at regular vaccination sites. The second group consisted of 132 subjects vaccinated under medical staff supervision at the emergency department at the University Clinical Centre Niš (Table 2). Patients who underwent vaccination under supervision were found to be statistically significantly older compared to the control group (*p* < 0.001) (Table 2). The place of residence was also found to be statistically significantly different between the groups (*p* = 0.010). Allergies, chronic conditions, diabetes, and other endocrinological diseases were found to be statistically significantly more frequent in the group of patients who underwent vaccination under supervision (Table 2).

**Table 2:** Demographic and clinical characteristics of the two studied subgroups

Parameter	Control		Supervised		*p* ^1^
	*n*	%	*n*	%	
Age^2^	39.4 ± 12.8		46.6 ± 11.3		<0.0012
Gender – male	35	37.6	41	31.8	0.445
Location					
City	24	25.3	16	12.1	0.010
Rural area	71	74.7	116	87.9	
Education level					
Primary	4	4.4	2	1.6	0.218
Secondary	48	53.3	58	45.3	
Post-secondary	14	15.6	19	14.8	
Tertiary	24	26.7	49	38.3	
Previous allergies	26	27.7	86	65.6	<0.001
Chronic diseases	23	24.2	54	40.9	0.009
Cardiovascular	14	14.7	28	21.2	0.215
Diabetes mellitus	2	2.1	12	9.1	0.047
Endocrinological	1	1.1	11	8.3	0.016
Kidney disorders	0	0	4	3.0	0.142
Oncological patients	1	1.1	2	1.6	1.000

^1^Chi-squared test.

^2^Student’s *t*-test.

Out of the total number of subjects enrolled in the study, only around 32% were ready to give data about the type of vaccine used, and a statistically significantly higher number of subjects willing to provide the information about the type of vaccine were those from the control group (Table 3). From the collected data, no significant difference between the two groups regarding the type of vaccine was found (Table 3).

**Table 3:** Vaccination profile among the interviewed subjects

Parameter	Total		Control		Supervised		*p*
	*n*	%	*n*	%	*n*	%	
Vaccinated	72	31.7	66	69.5^a^	6	4.5^b^	<0.001
Type of vaccine							
Pfizer	39	17.2	34	51.5	5	83.3	0.435
Sputnik V	2	0.9	2	3.0	0	0.0	
Sinopharm	29	12.8	28	42.4	1	16.7	
AstraZeneca	2	0.9	2	3.0	0	0.0	

^a^Significantly over-represented according to standardized adjusted residuals.

^b^Significantly under-represented according to standardized adjusted residuals.

Statistical analysis revealed that the GAD score was statistically significantly higher in subject who signed up for vaccination at the site where the medical staff supervision was available (Table 4). Among the BVS items, only BVS 1 and BVS 2 were found to be significantly different between the two studied groups (Table 4), while the BVS 3 and BVS 4, as well as total BVS, were almost identical.

**Table 4:** Total GAD and BVS scores, as well as scores distributed within the groups

Parameter	Total	Control	Supervised	*p* ^1^
GAD	5.93 ± 5.61	4.47 ± 5.01	6.98 ± 5.81	<0.001
BVS 1	4.88 ± 2.56	4.34 ± 2.66	5.27 ± 2.43	0.006
BVS 2	5.02 ± 2.51	4.40 ± 2.71	5.46 ± 2.27	0.001
BVS 3	2.94 ± 2.40	2.90 ± 2.52	2.96 ± 2.32	0.656
BVS 4	4.07 ± 2.19	4.39 ± 2.17	3.50 ± 2.16	0.126
Heart palpitation	3.98 ± 2.93	3.64 ± 2.87	4.02 ± 2.9	0.342
Unease	3.43 ± 3.02	3.03 ± 2.91	3.66 ± 3.11	0.165
Numbness	2.96 ± 2.8	2.54 ± 2.70	2.79 ± 2.77	0.586
Tingling	2.24 ± 2.33	1.75 ± 2.27	2.26 ± 2.58	0.209
Short breath	3.76 ± 3.32	3.07 ± 3.25	3.72 ± 3.40	0.242
Dizziness	3.43 ± 3.60	2.75 ± 3.48	3.40 ± 3.53	0.245
Feeling unreal	2.33 ± 2.79	1.86 ± 2.65	2.67 ± 3.10	0.103
Feeling insufficiently conscious	2.64 ± 3.13	2.16 ± 2.96	2.62 ± 3.07	0.312
Vertigo	3.12 ± 2.99	2.65 ± 3.06	3.30 ± 3.14	0.153
Hot flashes	2.97 ± 2.84	2.65 ± 2.94	2.41 ± 2.75	0.543
Palm sweating	2.28 ± 2.71	1.88 ± 2.42	2.16 ± 2.76	0.778
Stomach cramps	3.00 ± 3.14	2.84 ± 3.13	3.13 ± 2.88	0.359
Nausea	2.53 ± 2.8	2.18 ± 2.61	2.35 ± 2.52	0.512
Throat tightness	3.22 ± 3.39	2.67 ± 3.40	2.93 ± 3.17	0.361
Total BVS	15.62 ± 8.75	14.29 ± 9.08	16.25 ± 8.64	0.766

^1^Mann–Whitney test.

**Figure 1:**
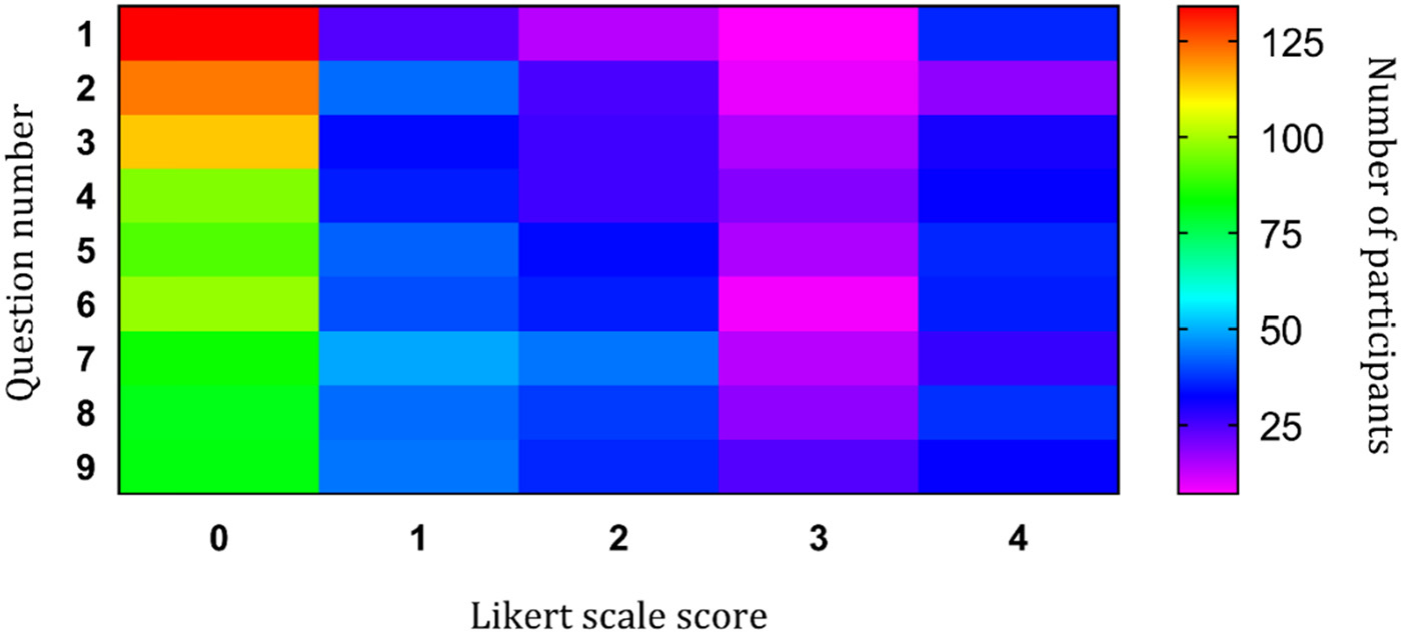
Heat map representing a number of subjects answering 0–4 (column) to a question of CAS (row). Red color indicates a greater number of subjects answering a given question and blue represents less answering a given question.

The total number of subjects, corresponding to a column in the heat map, indicates a response on a Likert scale (0–4) to the nine questions of CAS (Figure 1). As indicated by the heat map subjects reporting for vaccination mainly answered with “not at all” (score 0 on the Likert scale), followed by “rarely, for day or two” (score 1 on the Likert scale) and “almost every day” (score 4 on the Likert scale). The rarest answer score was 3 which is visible by the pale coulour in the Figure 1. It should provide a concise and precise description of the experimental results, their interpretation, as well as the experimental conclusions that can be drawn.

When the results of nine questions of CAS were compared between the two groups only, a significant difference in the frequency of their answers was found to be for the question concerning the recommendation for social distancing (Table 5). The answers that mostly accounted for this difference were the ones given by the subjects of the control group which were predominantly either “not at all” or “almost every day” (Table 5). Very close to a statistical significance between the groups were the answers for questions 1 and 7.

**Table 5:** Breakdown of scores for nine questions of COVID anxiety scale (CAS) given to subjects at different vaccination sites

Question	Supervised		Control		*p* ^1^
	*n*	%	*n*	%	
**1. I have avoided using public transport because of the fear of contracting coronavirus (COVID-19) (12 responses missing)**					
Likert score 0	62	70.5	72	56.7	0.056
Likert score 1	8	9.1	16	12.6	
Likert score 2	3	3.4	11	8.7	
Likert score 3	0	0.0^a^	7	5.5	
Likert score 4	15	17.0	21	16.5	
**2. I have checked myself for symptoms of coronavirus (COVID-19) (10 responses missing)**					
Likert score 0	54	60.7	68	53.1	0.535
Likert score 1	16	18.0	27	21.1	
Likert score 2	9	10.1	16	12.5	
Likert score 3	5	5.6	4	3.1	
Likert score 4	5	5.6	13	10.2	
**3. I have been concerned about not having adhered strictly to social distancing guidelines for coronavirus (COVID-19) (9 responses missing)**					
Likert score 0	54	58	64.4^a^	56	0.024
Likert score 1	16	11	12.2	22	
Likert score 2	9	9	10.0	17	
Likert score 3	5	2	2.2^b^	13	
Likert score 4	5	58	64.4^a^	56	
**4. I have avoided touching things in public spaces because of the fear of contracting coronavirus (COVID-19) (9 responses missing)**					
Likert score 0	42	46.7	54	42.2	0.667
Likert score 1	16	17.8	19	14.8	
Likert score 2	10	11.1	16	12.5	
Likert score 3	5	5.6	14	10.9	
Likert score 4	17	18.9	25	19.5	
**5. I have avoided going out to public places (shops, parks) because of the fear of contracting coronavirus (COVID-19) (10 responses missing)**					
Likert score 0	43	48.3	48	37.5	0.151
Likert score 1	15	16.9	27	21.1	
Likert score 2	14	15.7	19	14.8	
Likert score 3	2	2.2	13	10.2	
Likert score 4	15	16.9	21	16.4	
**6. I have read about news relating to coronavirus (COVID-19) at the cost of engaging in work (such as writing emails, working on word documents or spreadsheets) (9 responses missing)**					
Likert score 0	46	51.1	54	42.2	0.338
Likert score 1	19	21.1	21	16.4	
Likert score 2	11	12.2	24	18.8	
Likert score 3	2	2.2	6	4.7	
Likert score 4	12	13.3	23	18.0	
**7. I have checked my family members and loved one for the signs of coronavirus (COVID-19) (9 responses missing)**					
Likert score 0	43	47.8	41	32.0	0.140
Likert score 1	18	20.0	31	24.2	
Likert score 2	17	18.9	27	21.1	
Likert score 3	3	3.3	11	8.6	
Likert score 4	9	10.0	18	14.1	
**8. I have been paying close attention to others displaying possible symptoms of coronavirus (COVID-19) (10 responses missing)**					
Likert score 0	42	47.2	39	30.5	0.057
Likert score 1	16	18.0	27	21.1	
Likert score 2	11	12.4	27	21.1	
Likert score 3	4	4.5	14	10.9	
Likert score 4	16	18.0	21	16.4	
**9. I have imagined what could happen to my family members if they contracted coronavirus (COVID-19) (10 responses missing)**					
Likert score 0	42	46.7	40	31.3	0.174
Likert score 1	18	20.0	26	20.3	
Likert score 2	12	13.3	24	18.8	
Likert score 3	7	7.8	17	13.3	
Likert score 4	11	12.2	21	16.4	

^1^Chi-squared test.

^a^Significantly over-represented according to standardized adjusted residuals.

^b^Significantly under-represented according to standardized adjusted residuals.

There is a significant correlation between GAD and BVS 1 (*r* = 0.335, *p* < 0.001), with BVS 2 (*r* = 0.379, *p* < 0.001), and with BVS 3 (*r* = 0.283, *p* < 0.001), but there is no significant correlation with BVS 4 and total BVS (*r* = 0.073, *p* = 0.592, and *r* = 0.191, *p* = 0.171, respectively).

## 4 Discussion

Vaccination using different vaccines was carried out at different vaccination sites in Niš, Serbia. The vaccination process started early in January 2021 using all available vaccines at the time (Table 4). In 2021, at the University Clinical Center of Niš (Niš, Serbia), an action was created that offered vaccination to the population under the supervision of a team of experts: internists, anesthesiologists, and epidemiologists with the active participation of expert medical technicians from the fields of resuscitation, surgery, and internal medicine. The vaccination procedure was carried out in the Emergency Center of the University Medical Center Niš, with all the mentioned services available. Subjects’ application for the supervised vaccination procedure was voluntary, and subjects had the choice between currently available vaccines (Table 4).

Enrolled participants were aged between 18 and 68 years, with patients in the group that was vaccinated under medical staff supervision being significantly older (Table 2). The typical subject interviewed was female, from rural areas, with a secondary level of education, with potential previous allergies, and no chronic illness (Table 1). The type of subjects is potentially defined by the time of data collection (months after the vaccination process has started), which would make the people reporting for vaccination among the last ones willing to go with this procedure. Up to that point, sufficient reliable information has been provided to citizens through various media sources, compared to the information given at the outbreak of the pandemic [17], which was not an exception in Serbia [18]. Also, as suggested, excessive exposure to COVID-19-related news and information, especially negative and sensationalized content, might be linked to increased anxiety; however, these assumptions need to be further confirmed in further studies.

Interestingly, the media-given information did not significantly affect Serbian students’ tendency toward vaccination [19]. One should not overlook a low number of people willing to provide the information related to the type of vaccine that they were administered (Table 3). As suggested in Section 1, one of the factors affecting hesitance towards COVID-19 vaccination is associated with a mistrust of authorities and conspiracy theories [4].

In the group of patients vaccinated under medical staff supervision, a more significant number was affected by somatic illnesses (Table 2). Somatic illness makes a person prone to health-seeking behavior, careful observations (detection), and choice of somatic signals. A higher frequency of chronic illness among patients vaccinated under medical staff supervision (Table 2) can point to the attempt of those individuals to be close to a trained medical staff that could provide adequate medical support in the case of undesired effects of vaccination. Generally, patients suffering from chronic illnesses are reported to have vaccination rates approximately 10–20% lower than those of the general population [20]. Individuals with chronic conditions face a higher vulnerability to severe infections due to compromised immune systems. Additionally, frequent healthcare visits for treatments and check-ups expose them to potential sources of infection, especially in settings that involve close contact. Some healthcare institutions implemented various strategies, such as prioritization of vaccination programs and rigorous infection control measures, to minimize this [21].

Although there are different definitions of anxiety [22], some new concepts of anxiety involve the interpretation of interoceptive experiences and comparing them with the ones predicted by the limbic system [10,11]. On the other hand, a phrase “vaccine anxiety” is employed to characterize the fear, concern, or uneasiness that individuals may experience in anticipation of receiving a vaccine [23]. Levels of anxiety in subjects who opted for vaccination under supervision were found to be significantly increased compared to the group of subjects vaccinated at the control vaccination site (Table 3). Although GAD scores in the group of patients vaccinated under medical staff supervision are higher than in control, they are still around values that clinically are defined as mild anxiety. These lower GAD scores could be potentially related to an already made decision for vaccination, which might decrease one’s anxiety that has been increased during the decision-making process. Also, these results could be explained by the fact that subjects have collected sufficient information about the COVID-19 infection, ways of transmission, and vaccination process since the study was done during the second year of the pandemic. These general population results align with other publications where the values are in more than 70% of people below 5 [24].

As said GAD scores in the group of patients vaccinated under medical staff supervision could be defined as mild anxiety (score around 7; Table 3). This type of anxiety, according to cognitive and behavioral interpretations, is both useful and adaptive since it organizes activity and behavior. This signal anxiety is also adequate since it engages ego defense mechanisms, which are predetermined by the individual’s past and indicate future behavior patterns and potential development of psychopathology. At the same time, high anxiety causes inhibition and disturbs everyday behavior, which is both in line with cognitive and behavioral as well as with analytical standpoints. Thus, clinically significant anxiety has ruminant quality and inhibits behavior and decision-making, which would hypothetically prevent such subjects from coming to a vaccination site. On the other hand, those with lower anxiety levels (healthy anxiety) are capable of making decisions [22] that are in agreement with health preservation. In the present scenario, the vaccination process could also be interpreted as reassurance behavior and risk avoidance, i.e., risk-aversion behavior, i.e., direct avoidance of the death threat.

Although the study was conducted in 2021, anxiety related to COVID-19 and general anxiety was found to be slightly increased (Tables 4 and 5, Figure 1). Other studies reported lower or similar CAS scores [25,26] than the ones found here (Table 5 and Figure 1). This can be explained by the different study population, which differs from the general population, mainly with middle-level education (Table 1) studied herein. However, we should have in mind when interpreting the results that it could be affected by the time at which the subjects were interviewed (prior to vaccination or unrelated to vaccination).

The values of CAS were found to be significantly different between the two groups only in the dimension of the answer related to the social distancing recommendations (question 3). It is debatable what kind of answer subjects provide and the message behind the social distancing recommendation. One might answer a question from the standpoint “I’m thinking out distancing, which is recommended, I’m aware of the recommendation.” Also, it can reflect the inner worry for someone else we might encounter and whether the transmission between us is possible. Thus, this message carries a need for deeper analysis and observation.

The *p*-value, which is close to a statistical threshold, was obtained for the question,” I have been paying close attention to others displaying possible symptoms of coronavirus (COVID-19)” (Table 5). This can point to possible changes in attention, which, apart from self-screening for the anxiety symptoms, is from time to time shifted to others as they might be a potential source of infection. One might expect an increase in attention vigilance as a consequence of mild/moderate anxiety, which is frequently seen in patients with anxiety disorders seeking the source of danger [22,27]. The question is, is it possible that the obtained impulses/information are adequately integrated due to increased anxiety, or does this only serve for a rapid decrease of anxiety?

Body vigilance, consciously attending to internal cues, is a normal adaptive process, and the currently used scale is expected to measure the tendency of an individual to attend to panic-related body sensations [15]. The currently used BVS is demonstrated to be sensitive to determining body cues related to health- and safety-seeking behavior [28]. Changes in body vigilance are positively correlated with age [29], suggesting that the BVS should be increased in older subjects, such as those vaccinated under supervision. In the subjects vaccinated under medical staff supervision, BVS 1 and BVS 2 scores were statistically significantly increased (Table 4). However, total BVS was found not to be significantly different among the two studied groups (Table 4), which were proven to significantly differ in age (Table 2). This finding could be explained by the not-as-significant age difference that might accompany an increase in BVS.

The sum of scores on four questions on BVS, as well as the total BVS score, indicates that values in vaccinated patients are between those previously found in general non-anxious and anxious populations [28]. In a previous study of healthy subjects vaccinated for the influenza virus in Japan, the values of total BVS scores [30] were found to be very similar to the here detected ones (Table 4). The correlation of the GAD and BVS scores revealed that GAD is correlating significantly with the two (BVS 1 and BVS 2) items found to be significantly different between the two groups (Table 4). The two items in question could be also associated with increased attention vigilance since both are related to body scanning.

Body vigilance and the category of CAS in question 3 (I have been concerned about not having adhered strictly to social distancing guidelines for coronavirus [COVID-19]) was dimension more pronounced in the group of participants who voluntarily signed up to be vaccinated at the site under medical staff supervision. The traditional view is that attendance and sensitivity to somatic cues reflect withdrawal from the world of others, regression, egoistic position, and inability to be with others [31]. However, this factor from CAS points out that it might not be such. Sensorial perception precedes thoughts and cognitive constructs, and attending to one’s sensorial cues is the channel to understanding the sensorial cues of others. This communication channel precedes words and extends to interpersonal space, where the worlds overlap. It is ultimately a bodily awareness of this intertwining moment, a moment where gazes cross and perceptions overlap, the very factor that fosters our sensitivity towards other people. As said by Merleau-Ponty, “far from a world of detached egos, or one of mere objects, what we encounter through embodied perception is this crisscrossing of lateral, overlapping relations with other people, other creatures, and other things – an expressive space that existed between lived bodies. It is ultimately a body awareness of this intertwining that fosters our sensitivity towards other people” [32]. These notions are in line with the neural basis of empathy [33], with the theory of embodiment of experience [34], and with prediction error [11].

This can be potentially related to the fact that interoceptive signals mainly correspond to an actual medical illness and thus contribute to the sickness behavior [30], which is very clear in the present situation where the subjects did not suffer from COVID-19 infection nor some similar respiratory infection. Furthermore, it is suggested that chronic/repeated/intense stressors that disturb homeostasis, in our case psychological system, could cause dysfunction in interception. Supposedly, under this sort of stress, interceptive queue cues intensity, detection, monitoring, and feedback could alter [35]. In the present context, prolonged and intense anxiety following the pandemic outbreak might have led to a dysregulation and/or attenuation of body vigilance (decrease in interoception intensity detection), which occurred months before their visit to the vaccination site.

## 5 Conclusions

The results of the work will enable more efficient planning and organization of the epidemiological service, which during the pandemic period is faced with the great needs of the population and the need to work efficiently in organized periods of time. At a deeper level, we asked the question: What factors influence the behavior, motivation, and decision-making of an individual to accept a health-related procedure? Among the previously given explanations that impact the hesitance for vaccination, one could argue that anxiety and body vigilance might also play a part in this. Further studies are needed to determine what is in the bases of the drive for the noted anxiety and body vigilance in these subjects.
